# Factors associated with surgical site infection after ankle fracture fixation: a retrospective study in northern Sweden

**DOI:** 10.2340/17453674.2026.46313

**Published:** 2026-09-03

**Authors:** Sertaç TOPALHAFIZOGLU, Jacob STRÅLMARK, Rickard LUNDQVIST, Johan WÄNMAN, Grzegorz PIETZ

**Affiliations:** Department of Diagnostics and Intervention, Umeå University, Umeå, Sweden

## Abstract

**Background and purpose:**

Ankle fractures are common, and open reduction and internal fixation (ORIF) is standard treatment for unstable injuries. Surgical site infections (SSI) remain a significant clinical challenge. We aimed to estimate incidence and to identify patient- and treatment-related factors associated with SSI after ankle ORIF.

**Methods:**

In this retrospective cohort, we included all adults (≥ 18 years) who underwent ankle ORIF at 2 trauma centers in northern Sweden from 2015 to 2024. Data from medical records covered demographics, comorbidities, injury characteristics, surgical details, and peri/postoperative care. The primary outcome was incidence of SSI and the secondary outcome was independent factors associated with SSI. SSI was defined as an infection occurring at or near the surgical incision within 30 days after surgery or up to 1 year later in the presence of an implant.

**Results:**

We included 781 patients (mean age 54 years; 61% women). SSI occurred in 11%; 27% of infections underwent revision surgery, and 39 infections (43%) were categorized as fracture-related infection (FRI). Other complications included urinary tract infections (2.2%), pneumonia (0.8%), deep vein thrombosis (0.5%), and pulmonary embolism (0.4%). In adjusted analyses, higher odds of SSI were observed with smoking (aOR 2.3, 95% confidence interval [CI] 1.1–4.8, P = 0.02), initial external fixation (aOR 5.6, CI 1.7–17.6, P = 0.004), and longer operative time (OR per minute 1.0, CI 1.0–1.0, P = 0.004). The American Society of Anesthesiologists (ASA) class estimate suggested increased risk but was imprecise (aOR 9.7, CI 1.0–93.6, P = 0.049). Age, sex, and open fracture were not associated with SSI in the adjusted model.

**Conclusion:**

After ankle ORIF, SSI occurred in 11%. Smoking, initial external fixation, and longer operative time were associated with higher risk of infection, while ASA estimate was imprecise. These findings support perioperative risk mitigation (e.g., smoking cessation and operative efficiency) and motivate study of physiological risk (ASA).

Ankle fractures are among the most common intra-articular fractures in a weightbearing joint [1]. Given their high incidence and risk of long-term morbidity, ankle fractures represent a major socioeconomic burden [1-2,6]. Although open reduction and internal fixation (ORIF) effectively restores stability and alignment, it carries a considerable risk of complications. The most common are soft tissue problems such as surgical site infection (SSI), delayed healing, and implant failure, often associated with long surgical incisions, preoperative swelling, or impaired skin condition [1,7]. These factors may necessitate delayed surgery and increased infection risk. Clinical consequences include prolonged hospitalization, intravenous antibiotics, revision surgery, and, in severe cases, amputation [6]. Other reported complications are thromboembolic and cardiovascular events. Established risk factors include older age, smoking, diabetes, cardiovascular disease, and obesity, although findings remain inconsistent. Lynde et al. [7] found a higher rate of wound complications in patients with diabetes, whereas Aigner et al. [3] reported no such association. Overall, postoperative complication rates range between 19% and 40% [7–9].

Among these, SSI is the most frequent complication. SSI can impair fracture healing, delay rehabilitation, and may require reoperation [10]. Reported factors associated with SSI include advanced age, high body mass index (BMI), open fracture, smoking, diabetes, high-energy trauma, prolonged operative time, and institutional living [11-16]. In more severe cases, infection may progress to fracture-related infection (FRI). SSI is defined as an infection occurring at or near the surgical incision within 30 days after surgery or up to 1 year later in the presence of an implant; in contrast, FRI refers to infection involving the fracture site and bone itself [17].

Most prior studies are limited by small datasets and lack of multivariable models including both clinical and radiographical parameters. Thus, the interplay between fracture characteristics, classification, and comorbidities in predicting SSI remains unclear. Our study aims to estimate the incidence of SSI and to identify independent factors associated with postoperative infection after ORIF for ankle fractures.

## Methods

### Study design and setting

This retrospective cohort study collected data from 2 centers within region Västerbotten, Sweden: Umeå University Hospital (Level I trauma center) and Skellefteå Hospital (Level II trauma center). The study encompassed all adult patients (≥ 18 years) who underwent ORIF for acute ankle fractures between January 1, 2015, and December 31, 2024. This study was reported according to the Strengthening the Reporting of Observational Studies in Epidemiology (STROBE) guideline.

### Participants

Patients were eligible for inclusion if they had undergone surgical treatment for an acute ankle fracture. Exclusion criteria included: age < 18 years, pathological fractures, delayed presentation (> 14 days post-injury), polytrauma, tibial plafond fractures, and residence outside Västerbotten County. An opt-out design was utilized for obtaining informed consent, whereby eligible patients were contacted via letter prior to the study’s initiation.

### Data collection and variables

Data was collected from electronic medical records using a standardized protocol. Extracted variables included demographic and clinical information such as age, sex, BMI, tobacco and alcohol use, and relevant comorbidities, including diabetes mellitus, dementia, corticosteroid use, peripheral arterial disease, and chronic venous insufficiency. Injury characteristics were recorded, including the mechanism of injury, classified as low- or high-energy, fracture classification according to the AO/OTA system, presence of dislocation or subluxation, and soft tissue condition. Surgical details were documented, including time from injury to definitive surgery, operative duration, surgical approach, fixation method, American Society of Anesthesiologists Physical Status (ASA) classification, use of preoperative and perioperative antibiotics, and the application of external fixation. Postoperative care variables were extracted from medical records and included the use of thromboprophylaxis, length of hospital stay, urinary tract infections, pneumonia, deep vein thrombosis, and pulmonary embolism.

Radiographic fracture classification was performed according to the AO/OTA system using anteroposterior, lateral, and mortise views. All patients were managed according to local standardized treatment protocols, which included immobilization prior to surgery, administration of intravenous prophylactic antibiotics (cloxacillin 2 g preoperatively, with a repeat dose after 2 and 6 h if the surgery time was over 2 h or clindamycin 600 mg), and low-molecular-weight heparin (LMWH) for thromboprophylaxis.

### Outcomes

The primary outcome was the occurrence of surgical site infections (SSIs). SSI was defined as a culture-verified infection at the surgical site, confirmed by positive microbiological growth from wound or deep tissue samples obtained during postoperative assessment, reoperation, or clinical signs of infection treated with antibiotics. All data was collected from the patients’ medical records by 2 evaluators (specialists in orthopedics). No inter-rater reliability was performed on the data collected. FRI was classified according to the consensus on definition reported by Metsemakers et al. [17]. FRI comprises the subset of patients within the SSI cohort who met the FRI diagnostic algorithm. All of these patients were evaluated against confirmatory inclusion criteria, such as the presence of a fistula, purulence, and positive deep cultures from both tissue samples and sonication. Secondary outcome was independent factors associated with SSI.

### Statistics

Descriptive statistics were used to summarize patient characteristics. Univariate analyses (chi-square test for categorical variables and t-test for continuous variables) were conducted to assess associations with SSI. Normally distributed variables are presented as mean with standard deviation (SD), while non-normally distributed variables are presented as median with interquartile range (IQR). Normality was assessed using histogram and Q–Q plots. Cramér’s V was used for the effect size analysis. Multivariable logistic regression was used to identify independent risk factors for SSI. Odds ratios (ORs) with 95% confidence intervals (CIs) were reported. Analyses followed a complete-case approach; missing data was not imputed. A P value < 0.05 was considered statistically significant. All data was analyzed by IBM SPSS^®^ version 29.0 (IBM Corp, Armonk, NY, USA).

### Ethics, data sharing plan, funding, use of AI, and disclosures

This retrospective cohort study was approved by the Swedish Ethical Review Authority (approval number 2024-02226-01) and conducted in accordance with the principles of the Declaration of Helsinki. Study registration was not applicable due to the retrospective design.

The data that supports the findings of this study is available from the corresponding author upon reasonable request.

Artificial intelligence was used for language editing and proofreading of the manuscript using ChatGPT. This work was supported by funding from the Region Västerbotten through the ALF agreement. This research was not supported by grants from any public, commercial, or not-for-profit funding agencies.

The authors declare no conflicts of interest. Complete disclosure of interest forms according to ICMJE are available on the article page, doi: 10.2340/17453674.2026.46313

## Results

### Study population

Of 839 eligible patients, 58 opted out after being contacted via letter, resulting in a final study cohort of 781 patients who underwent surgical treatment for ankle fractures between 2015 and 2024 and were included in the study (Figure). The cohort consisted of 479 women (61%) and 302 men (39%), with a mean age of 54 (SD 18) years. The BMI mean was 28 (SD 5.3). 26% of patients were classified as ASA 1, 45% as ASA 2, 15% as ASA 3, and 0.9% as ASA 4. Most fractures were classified as Weber B (72%), followed by Weber C (23%) and Weber A (5%). Based on the AO classification, the most common fracture types were 44b3 (42%), 44b2 (19%), and 44c2 (13%) (Table 1). Among patients who developed SSI, the most administered antibiotic was cloxacillin 2 g (75%), followed by clindamycin 600 mg (7%). The most common prophylactic dosing regimen among patients with SSI was a single dose (36%), followed by 3 doses (27%) (Table 2).

**Figure:**
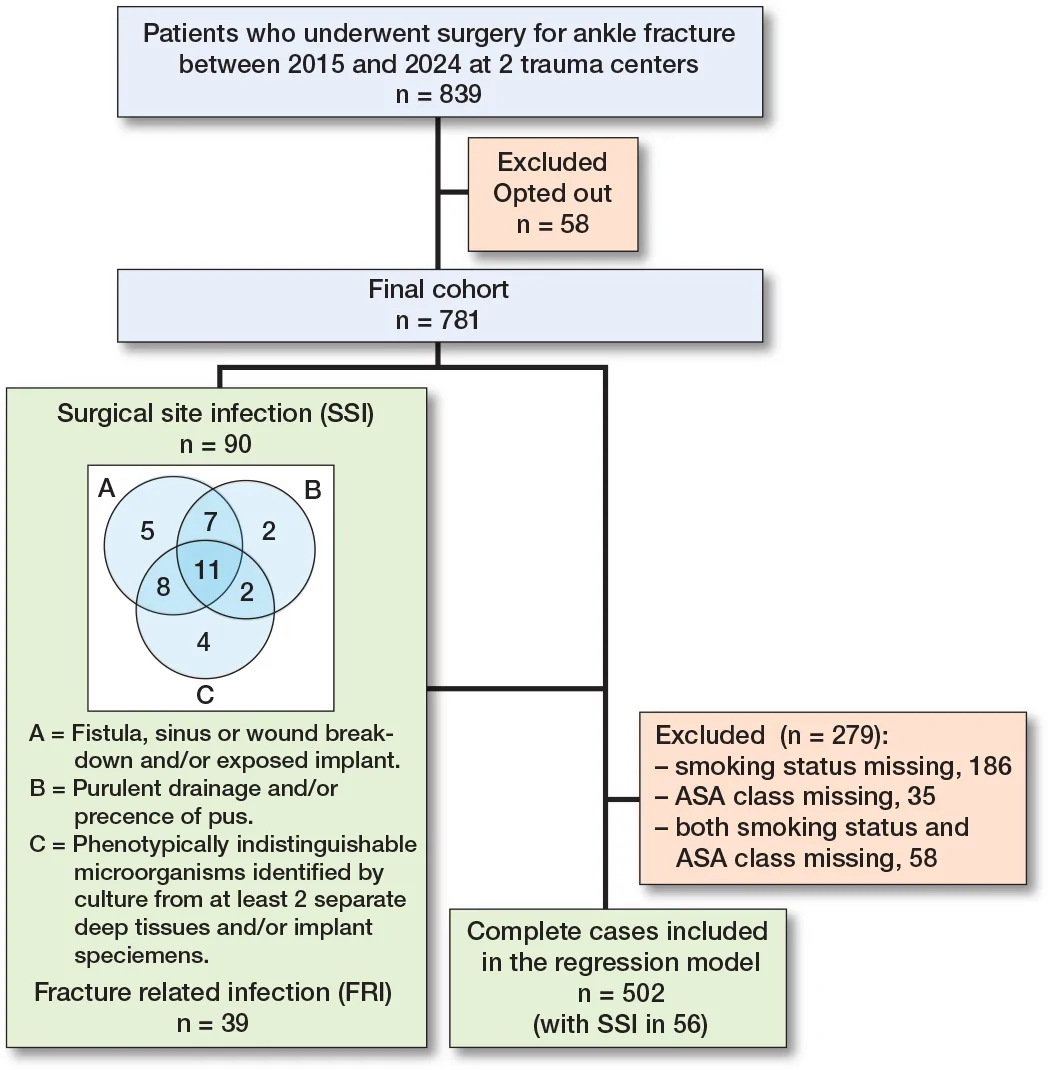
Flowchart of the study.

**Table 1:** Participants’ characteristics and preoperative data (N = 781). Values are count (%) unless otherwise specified

Age at surgery, mean (SD)	54 (18)
Sex	
Female	479 (61)
Male	302 (39)
Body mass index, mean (SD)	28 (5)
Functional status: ASA classification	
1	208 (27)
2	353 (45)
3	121 (16)
4	7 (0.9)
Fracture type: Weber classification	
A	38 (4.9)
B	562 (72)
C	181 (23)
Fracture type: AO classification	
44a1	1 (0.1)
44a2	32 (4.1)
44a3	7 (0.9)
44b1	84 (11)
44b2	150 (19)
44b3	328 (42)
44c1	53 (6.8)
44c2	101 (13)
44c3	25 (3.2)
Number of fractured malleoli	
1	181 (23)
2	262 (34)
3	338 (43)
Subluxation/dislocation	409 (52)
Trauma type	
Low energy	742 (95)
High energy	39 (5.0)
Open fracture	18 (2.3)
Swollen	769 (99)
Blisters	51 (6.5)
Wound	60 (7.7)
Preoperative antibiotics	655 (84)
Foot pump	115 (15)
Initial external fixation	38 (4.9)

ASA = American Society of Anesthesiologists.

**Table 2:** Operative and postoperative data for participants with surgical treatment for an acute ankle fracture (N = 781). Values are count (%) unless otherwise specified

Days from injury to surgery, median (IQR)	1.4 (0.8–4.0)
Operation time (minutes), mean (SD)	92 (42)
Lateral malleolus fixation	695 (89)
Medial malleolus fixation	440 (56)
Posterior malleolus fixation	55 (7.0)
Syndesmotic fixation	404 (52)
Wound closure	
Suture	730 (94)
Agraffe	47 (6.0)
Intracutaneous suture	4 (0.5)
Postoperative thromboprophylaxis	780 (100)
Postoperative antibiotics	180 (23)
Weightbearing	341 (44)
Post surgery stays (days), median (IQR)	1.2 (0.9–2.3)
Hospital stay (days), median (IQR)	2.9 (1.8–5.0)
Postoperative complications	
Deep venous thrombosis	4 (0.5)
Pulmonary embolism	3 (0.4)
Pneumonia	6 (0.8)
Urinary tract infection	17 (2.2)
Surgical site infection (SSI)	90 (12)
Implant related complications	212 (28)
Symptomatic implant ^**a**^	150 (19)
Implant failure ^**b**^	19 (2.4)
Inadequate fixation ^**c**^	20 (2.6)
Implant infection	28 (3.6)
Revision surgery	214 (27)

a Defined as implant causes discomfort, pain, or other problematic symptoms for the patient.

b Defined as mechanical failure of the implant.

c Defined as poor technique or malalignment of the implant.

### Occurrence of SSI

SSI occurred in 90 patients (11%). Other complications included urinary tract infections (2.2%), pneumonia (0.8%), deep vein thrombosis (0.5%), and pulmonary embolism (0.4%). Implant-related complications were observed in 28% of patients, with symptomatic implants being the most common (19%). 27% of patients required revision surgery (see Table 2). 39 of the 90 patients (43%) were categorized as having FRI.

### Primary outcome

Patients who developed SSI were older, with a mean age difference of 6 years (CI 1.8–9.8), and more frequently had ASA class 3–4 (Cramér’s V 0.18). Open fractures were associated with increased odds of SSI (OR 4.0, CI 1.5–11.1), as were preoperative skin wounds (OR 2.9, CI 1.6–5.5), subluxation/dislocation at presentation (OR 1.8, CI 1.1–2.8), and initial external fixation (OR 5.8, CI 2.9–11.6) (Table 3).

**Table 3:** Association between SSI and health-related factors among patients (N = 781), who underwent surgical treatment for ankle fracture between 2015 and 2024

Characteristic Classification	SSI n = 90	No SSI n = 691	Difference, OR, or effect size (CI)
Age, mean (SD)	59 (18)	53 (18)	6.0 (1.8–9.8)
Sex			
Male	29 (32)	273 (40)	1.4 (0.9–2.2)
Female	61 (68)	418 (61)	
BMI, mean (SD)	28 (6)	28 (5)	0.5 (–0.8 to 1.8)
Functional status (ASA class)			
1	10 (11)	198 (29)	0.175 ^**c**^
2	40 (44)	312 (45)	
3	25 (28)	96 (14)	
4	3 (3.3)	4 (0.6)	
Fracture type			
Weber A	2 (2.2)	36 (5.2)	0.050 ^**c**^
Weber B	69 (77)	493 (71)	
Weber C	19 (21)	162 (23)	
Open fracture			
No	84 (93)	679 (98)	4.0 (1.5–11.0)
Yes	6 (6.7)	12 (1.7)	
Swollen			
No	1 (1.1)	12 (1.7)	1.6 (0.2–12.2)
Yes	89 (99)	679 (98)	
Blisters			
No	81 (90)	649 (94)	1.7 (0.8–3.7)
Yes	9 (10)	42 (6.1)	
Wound			
No	75 (83)	647 (94)	2.9 (1.6–5.5)
Yes	15 (17)	44 (6.4)	
Subluxation/dislocation pre			
No	32 (36)	341 (49)	1.8 (1.1–2.8)
Yes	58 (64)	350 (51)	
Initial external fixation			
No	75 (82)	668 (97)	5.8 (2.9–11.6)
Yes	15 (17)	23 (3)	
Days from injury to surgery, mean (SD)	4 (4)	3 (4)	1.6 (0.7–2.4)
Operation time, minutes, mean (SD)	108 (49)	90 (41)	18 (8.7–27.0)
Comorbidities			
Smoking (n = 537) ^**d**^			
No	41 (46)	411 (77)	29 (1.6–5.3)
Yes	19 (21)	66 (10)	
Alcohol/drug consumption			
No	80 (89)	646 (93)	1.8 (0.9–3.7)
Yes	10 (11)	45 (7)	
Diabetes			
No	78 (87)	630 (91)	1.6 (0.8–3.1)
Yes	12 (13)	61 (8.8)	
Immunosuppressive therapy/corticosteroids			
No	77 (86)	665 (96)	4.3 (2.1–8.8)
Yes	13 (14)	26 (3.8)	
Peripheral arterial disease/venous insufficiency			
No	84 (93)	685 (99)	8.2 (2.6–25.9)
Yes	6 (6.7)	6 (0.9)	
Dementia			
No	88 (98)	669 (97)	0.7 (0.2–3.0)
Yes	2 (2.7)	22 (3.2)	
Preinjury immobility (n = 780) ^**d**^			
No	82 (92)	647 (94)	1.3 (0.6-2.9)
Yes	7 (7.8)	44 (6.4)	
Post-surgery stay, days, mean (SD)	3.1 (2.8)	2.0 (2.4)	1.2 (0.6–1.7)
Total hospital stay, days, mean (SD)	6.3 (5.2)	3.7 (3.8)	2.6 (1.7–3.4)
Weightbearing (n = 780) ^**d**^			
No	57 (63)	382 (55)	0.7 (0.5–1.1)
Yes	33 (37)	308 (45)	

ASA = American Society of Anesthesiologists; BMI = body mass index; CI = 95% confidence interval; OR = odds ratio.

a Independent t-test.

b Chi-squared test.

c Cramér’s V as effect size.

d Missing data; the prevalence is based on complete cases for the specific variable in each analysis.

Patients with SSI also had longer injury-to-surgery intervals, with a mean difference of 1.6 days (CI 0.7–2.4), and longer operative duration, with a mean difference of 18 min (CI 8.7–27.0). Smoking (OR 2.9, CI 1.6–5.3), immunosuppressive or corticosteroid therapy (OR 4.3, CI 2.1–8.8), and peripheral arterial disease or venous insufficiency (OR 8.2, CI 2.6–25.9) were also associated with increased odds of SSI. Postoperatively, patients with SSI had longer postoperative stays (mean difference 1.2 days, CI 0.6–1.7) and total hospital stays (mean difference 2.6 days, CI 1.7–3.4).

### Multivariable predictors of SSI

In the multivariable logistic regression analysis, several independent risk factors for SSI were identified (Table 4). Smoking was associated with more than twice the odds compared with non-smokers (OR 2.3; CI 1.1– 4.8; P = 0.02). Each additional minute of operative time was associated with a small but statistically significant increase in infection risk (OR 1.01, CI 1.00–1.02; P = 0.004).

**Table 4:** Associated factors (potential predictors) of SSI among patients who underwent surgical treatment for ankle fracture between 2015 and 2024

Variables	Adjusted ^a^ OR (CI)	P value
Age	1.0 (0.9–1.0)	0.8
Sex (ref. male)	0.9 (0.5–1.9)	0.8
Functional status (ref. ASA 0/1)		
ASA 2	1.8 (0.7–4.5)	0.2
ASA 3	3.0 (1.0–9.0)	0.052
ASA 4	9.7 (1.0–93.6)	0.049
Open fracture (ref. no)	0.8 (0.2–3.8)	0.8
Wound (ref. no)	1.6 (0.7–4.0)	0.3
Initial external fixation (ref. no)	5.6 (1.7–17.6)	0.004
Time from injury to surgery (continuous, days)	1.0 (0.9–1.1)	0.9
Operation time (continuous, min)	1.01 (1.00–1.02)	0.004
Smoking (ref. no.)	2.3 (1.1–4.8)	0.02
Immunosuppressive therapy/corticosteroids (ref. no.)	1.9 (0.7–5.6)	0.2
Peripheral arterial or venous insufficiency (ref. no).	4.4 (0.9–21.3)	0.07

ASA = American Society of Anesthesiologists classification; CI = 95% confidence interval; OR = odds ratio.

For the logistic regression models (univariable and multivariable), the reference categories were: male sex, ASA 1, no wound, closed fracture, no subluxation/dislocation, no initial external fixation, no smoking, no immunosuppressive therapy/corticosteroids, no peripheral arterial disease and no venous insufficiency. The adjusted model includes 502 complete cases; missing cases are due to incomplete data on smoking (n = 244) and ASA (n = 93).

a Multivariable model.

Higher ASA classification was also associated with SSI. Patients classified as ASA 4 had increased odds over those classified as ASA 1 (OR 9.7, CI 1.0–93.6; P = 0.049). Furthermore, the use of initial external fixation was strongly associated with SSI (OR 5.6, CI 1.7–17.6; P = 0.004). Other variables, including age, sex, open fracture and time from injury to surgery, were not significant in the adjusted model.

## Discussion

Our study primarily aimed to determine the incidence of SSI and secondarily to identify factors associated with SSI. SSI occurred in 11% of cases and smoking, initial external fixation, and longer operative time were independently associated with higher odds of SSI, while ASA class showed a large but imprecise estimate.

The SSI rate of 11% is comparable to previously reported rates ranging between 5% and 15% [7-9]. Moreover, more than one-quarter of patients (27%) required revision surgery, underscoring the substantial clinical burden of complications in this patient population.

In the multivariable analysis, smoking, higher ASA class, prolonged operative time, and the use of initial external fixation emerged as independent factors for SSI. While smoking, systemic comorbidity, and operative time have consistently been identified as predictors in earlier studies [10-16], the finding that temporary external fixation independently increases the risk of infection appears to be novel and warrants particular attention. External fixation is typically reserved for cases with severe soft tissue compromise, open fractures, or gross dislocation, and has traditionally been regarded as a temporizing measure rather than a direct risk factor. Our findings suggest that, independent of other variables, the use of external fixation may itself contribute to infection risk. To our knowledge, previous literature has not identified this association, making this an important contribution. The mechanism may relate to additional skin penetration, colonization along pin tracts, or the selection of patients with particularly vulnerable soft tissues. Nevertheless, it raises the question of whether external fixation, beyond serving as a marker for severe injury, could act as a modifiable risk factor for infection and thus warrants further prospective investigation.

Smoking remains a well-established, modifiable risk factor in orthopedic surgery [11-14]. In our cohort, smokers had more than twice the odds of developing SSI compared with non-smokers, in line with prior literature showing up to a fivefold increased risk [14]. However, no evidences exist for cessation postoperatively. Similarly, ASA classification, as a surrogate for overall health status, was a strong predictor of infection risk, with ASA 4 patients showing nearly tenfold higher odds than ASA 1, consistent with prior reports [15,16]. Nevertheless, the findings warrant cautious interpretation because the wide confidence intervals reflect limited precision of the estimates.

Prolonged operative time was also strongly associated with SSI, which likely reflects increased tissue handling, complex fracture patterns, or intraoperative challenges. This emphasizes the need for efficient surgical technique and careful preoperative planning [12,18-21]. In contrast to other studies, timing of surgery was not associated with SSI [22].

Age was significant in the univariable analysis but did not remain significant after multivariable adjustment, whereas diabetes was not significant in the univariable analysis and was therefore not included in the multivariable model. While several studies have reported associations between diabetes and postoperative complications [7,23-24], our findings are in line with others that did not confirm this link [3]. It is possible that the effects of age and diabetes are mediated through more global measures of health status, such as ASA classification, or through vascular comorbidity.

A substantial proportion of patients with SSI (43%) fulfilled the criteria for fracture-related infection (FRI), underscoring the clinical severity of infectious complications in this cohort. Compared with superficial SSI, FRI was associated with earlier revision surgery and more extensive interventions, including implant removal and, in one case, amputation. These findings highlight the progressive nature of deep infections involving the fracture site and emphasize the importance of early identification and prompt management to prevent transition from superficial SSI to established FRI. Given the considerable morbidity and resource utilization associated with FRI, preventive strategies targeting modifiable factors are particularly relevant.

### Strengths

This is a large cohort size, combined with standardized treatment protocols from 2 trauma centers, allowing robust assessment of both patient- and treatment-related factors.

### Limitations

We may have underreporting of minor complications or incomplete documentation. Furthermore, although we identified external fixation as a novel independent risk factor, causality cannot be established in this study design, and prospective validation is needed. An additional limitation is the risk that subclinical infections may have been missed, as these often present without clear symptoms and can therefore be difficult to identify in a retrospective chart review.

### Conclusion

We found a SSI incidence of 11%. In adjusted analyses, smoking, initial external fixation, and prolonged operative time were associated with higher infection risk, whereas the ASA estimate suggested increased risk but was imprecise.

*In perspective,* these findings support targeted perioperative risk-mitigation (e.g., smoking cessation and operative efficacy) to reduce SSI and improve outcomes.

### Supplementary data

Supplemenatry Table S1 is available as supplementary data on the article page, doi: 10.2340/17453674.2026.46313

## Supplementary Material



## Notes

JW and GP designed the study. ST, JS, and RL collected the data. ST drafted the manuscript. GP performed data processing and statistical analyses. All authors contributed to the revision and approved the final manuscript.

Handling co-editors: Bart Pijls and Robin Christensen

*Acta* thanks Nader Maai, Fintan Moriarty and Marc Gerard Romijn for help with peer review of this manuscript.
