# Supplementary material for: Factors associated with surgical site infection after ankle fracture fixation: a retrospective study in northern Sweden
Source: Acta Orthop. 2026 Sep 3;97:623–8. doi: 10.2340/17453674.2026.46313 (PMC13543151; doi:10.2340/17453674.2026.46313)
Supplement: Supplementary file 1 [file ActaO-97-46313-s1.pdf]

Supplementary data

Supplementary Table 1. Associated factors (potential predictors) of SSI among patients who underwent surgical treatment for ankle fracture between 2015 and 2024

Variables	Unadjusted [a] OR (CI)	P value	Adjusted [b] OR (CI)	P value
Age	1.0 (1.0–1.0)	0.005	1.0 (0.9–1.0)	0.8
Female sex (ref. male)	1.4 (0.9–2.2)	0.2	0.9 (0.5–1.9)	0.8
Functional status (ref. ASA 0/1)				
ASA 2	2.5 (1.2–5.2)	0.01	1.8 (0.7–4.5)	0.2
ASA 3	5.2 (2.4–11)	< 0.001	3.0 (1.0–9.0)	0.052
ASA 4	15 (2.9–75)	0.001	9.7 (1.0–94)	0.049
Open fracture (ref. no.)	4.0 (1.5–11)	0.006	0.8 (0.2–3.7)	0.8
Wound (ref. no.)	2.9 (1.6–5.5)	< 0.001	1.6 (0.6–4.0)	0.3
Initial external fixation (ref. no.)	5.8 (2.9–12)	< 0.001	5.6 (1.7–18)	0.004
Time from injury to surgery (continuous, days)	1.0 (1.0–1.1)	< 0.001	1.0 (0.9–1.1)	0.9
Operation time (continuous, min)	1.0 (1.0–1.0)	< 0.001	1.0 (1.00–1.0)	0.004
Smoking (ref. no.)	2.9 (1.6–5.3)	< 0.001	2.3 (1.1–4.9)	0.02
Immunosuppressive therapy/corticosteroids (ref. no.)	4.3 (2.1–8.7)	< 0.001	1.9 (0.6–.5.5)	0.2
Peripheral arterial or venous insufficiency (ref. no.)	4.2 (1.1–16)	0.02	4.3 (0.9–21)	0.07

ASA= American Society of Anesthesiologists classification; CI = 95% confidence interval; OR = odds ratio.

For the logistic regression models (univariable and multivariable), the reference categories were: male sex, ASA 1, no wound, closed fracture, no subluxation/dislocation, no initial external fixation, no smoking, no immunosuppressive therapy/corticosteroids, no peripheral arterial disease, and no venous insufficiency.

[a] Univariable model.

[b] Multivariable model.
